# Role of Dietary Long-Chain Polyunsaturated Fatty Acids in Infant Allergies and Respiratory Diseases

**DOI:** 10.1155/2012/730568

**Published:** 2012-08-28

**Authors:** Lynette P. Shek, Mary Foong-Fong Chong, Jia Yi Lim, Shu-E Soh, Yap-Seng Chong

**Affiliations:** ^1^Department of Paediatrics, Yong Loo Lin School of Medicine, National University of Singapore, National University Health System, Tower Block, Level 12, 1E Kent Ridge Road, Singapore 119228; ^2^Singapore Institute for Clinical Sciences, Agency for Science, Technology and Researsh (A*STAR), Brenner Centre for Molecular Medicine, Singapore 117609; ^3^Saw Swee Hock School of Public Health, National University of Singapore, Singapore 117597; ^4^Department of Obstetrics and Gynaecology, Yong Loo Lin School of Medicine, National University of Singapore, National University Health System, Singapore 119228

## Abstract

Maternal nutrition has critical effects on the developing structures and functions of the fetus. Malnutrition during pregnancy can result in low birth weight and small for gestational age babies, increase risk for infection, and impact the immune system. Long-chain polyunsaturated fatty acids (PUFAs) have been reported to have immunomodulatory effects. Decreased consumption of omega-6 PUFAs, in favor of more anti-inflammatory omega-3 PUFAs in modern diets, has demonstrated the potential protective role of omega-3 PUFAs in allergic and respiratory diseases. In this paper, we examine the role of PUFAs consumption during pregnancy and early childhood and its influence on allergy and respiratory diseases. PUFAs act via several mechanisms to modulate immune function. Omega-3 PUFAs may alter the T helper (Th) cell balance by inhibiting cytokine production which in turn inhibits immunoglobulin E synthesis and Th type 2 cell differentiation. PUFAs may further modify cellular membrane, induce eicosanoid metabolism, and alter gene expression. These studies indicate the benefits of omega-3 PUFAs supplementation. Nevertheless, further investigations are warranted to assess the long-term effects of omega-3 PUFAs in preventing other immune-mediated diseases, as well as its effects on the later immunodefense and health status during early growth and development.

## 1. Introduction

The *in utero* environment which is extremely susceptible to maternal influence plays an important role in the fetal growth and development. Maternal metabolic and endocrine function placental function as well as maternal diet can have critical effects on various aspects of developing structures and functions of the fetus [1]. Maternal malnutrition during pregnancy has been shown to result in low birth weight and delivery of small for gestational age (SGA) babies [2], as well as increased risk for neonatal infection [3, 4]. During infancy, malnutrition can greatly impact the developing immune system functionally and permanently [5, 6]. Changes in dietary patterns with urbanization have been reported to decrease immune tolerance, thus contributing to the rising rates of the immune disease [7]. Besides oligosaccharides, folate, and other vitamins which have been documented to play a role in the immune function [7, 8], dietary lipids have also been reported to have immunomodulatory effects [8], and the immunoactive properties of the polyunsaturated fatty acids (PUFAs) have been utilized in a variety of clinical settings [9, 10]. In this paper, we focus our review on how dietary polyunsaturated fatty acids consumption during pregnancy and early childhood may affect the outcomes of allergy and respiratory diseases in the offspring. 

## 2. Polyunsaturated Fatty Acids (PUFAs): Sources and Intakes

Polyunsaturated fatty acids (PUFAs) consist of two main groups of essential fatty acids: omega-3 (*n*-3) and omega-6 (*n*-6) [11, 12]. The simplest forms of the omega-3 and omega-6 PUFAs are alpha-linolenic acid (ALA) and linoleic acid (LA), respectively [11, 12]. The omega-3 fatty acid ALA can be metabolized into longer and more desaturated eicosapentaenoic acid (EPA) and docosahexaenoic acid (DHA) while the omega-6 fatty acid LA can be synthesized into long-chain arachidonic acid (AA) [11]; however, the conversion rates are usually low, ranging from 1 to 10% [13–16]. The conversion also varies depending on common polymorphisms in the fatty acid desaturate (FADS) gene cluster, which can result in different amounts of EPA, DHA, and AA being formed in different individuals [17, 18]. It has been reported that conversion rates are lower in infants than adults and insufficient conversion of ALA to EPA and further to DHA, particularly in premature infants, will have adverse effects on visual and neural development [19, 20].

Significant quantities of LA are found in vegetable oils such as corn, sunflower, and soybean and peanut oils as well as in products made from these oils such as margarines [11, 12]. Sources for ALA are green plant tissues, flaxseed, walnut, beechnut, butternuts, chia seeds, canola, and soy [11]. In most Western diets, as much as 98% of LA and ALA contribute to dietary PUFAs intake, with LA intake being in excess of that of ALA [12]. The intake of LA in the Western diet has increased markedly over the second half of the twentieth century, following the introduction and increased consumption of cooking oils and margarines, whereas ALA intake did not change much over this time [12]. The changed pattern of LA consumption has resulted in a marked increase in the ratio of omega-6 to omega-3 PUFAs in the diet, with the current ratio being between 5 and 20 in most Western populations [21]. The increased intake of the omega-6 PUFA linoleic acid has been claimed to be causally related to increased prevalence and incidence of atopic diseases in children [22, 23].

In developing countries, where energy and fat intake is low, LA and ALA would be preferentially used for energy expenditure rather than to synthesize EPA + DHA and AA [11]. In addition, micronutrients such as iron, zinc, vitamin B6, and vitamin E are required for the conversion of ALA and LA to EPA + DHA, resulting in lower levels of EPA + DHA and AA in these nutrient deficient populations [24].

## 3. Effects of Dietary PUFAs in Allergy and Respiratory Diseases

With the decline in the consumption of omega-3 PUFAs in favor of more proinflammatory omega-6 PUFAs in modern diets, numerous studies have demonstrated the potential protective role of omega-3 PUFAs in allergic diseases [7, 12]. Omega-3 PUFAs can be obtained from both fish and fish oils, and these fatty acids may oppose the actions of omega-6 PUFAs [12]. Kremmyda et al. [12] have done a comprehensive systematic review of the effects of early exposure to omega-3 PUFAs on atopy risk in infants and children. According to the review, maternal fish intake during pregnancy has been consistently demonstrated to have protective effects on atopic or allergic diseases in infants and children, such that maternal fish intake was inversely associated with eczema (adjusted odds ratio (OR): 0.75; 95% confidence interval (CI): 0.57, 0.98), asthma (OR: 0.20; 95% CI: 0.06, 0.65), and sensitization to food and dust mites [25–28]. However, this is not the case for the effects of fish intake during infancy or childhood on atopic outcomes, namely, eczema, hay fever, and asthma. The effects have been inconsistent, although the majority of the studies reported protective effects. This variation could be attributed to the fact that these studies had different designs, control of confounders, and exposures as well as different assessments on the study outcomes [12].

Fish oil supplementation during pregnancy and lactation have demonstrated higher provision of omega-3 PUFAs to the offspring and that early fish oil provision was associated with immunologic changes, such as increased cytokine production in cord blood [29–33]. These studies suggest that there are clinical effects of early fish oil provision including reduced sensitization to common food allergens (egg, milk, and wheat) and reduced prevalence and severity of atopic dermatitis (adjusted OR: 0.22; 95% CI: 0.06, 0.81) in the first year of life. On the other hand, a study on 706 infants in Australia demonstrated that high-dose omega-3 PUFAs supplementation of 900 mg/day in pregnancy did not reduce the overall incidence of immunoglobulin E (IgE) associated food allergy in the first 12 months of life, although omega-3 PUFAs supplementation lowered the incidence of atopic eczema and egg sensitization [34]. Fish oil supplementation during infancy or childhood has also shown to result in higher omega-3 PUFAs status in infants or children and that fish oil provision may be associated with immunologic changes in the blood [35–43]. However, it is not clear whether these are of clinical significance and if these changes persist as other factors come into play. 

Although the majority of the studies have focused on the role of omega-3 PUFAs on allergy, few studies have examined the role of dietary PUFAs supplement in respiratory diseases. As shown in Table 1, dietary supplementation of DHA and AA is associated with delayed onset and reduced risk of upper respiratory infection and asthma, allergic rhinitis, allergic conjunctivitis, atopic dermatitis up to three years of age [44], and lower incidence of bronchiolitis in the first year of life [45], as well as fewer illness episodes and lower incidence of respiratory illness [46]. However, in colostrum samples fed to 580 infants, higher concentrations of AA, DHA, and total omega-3 PUFAs were associated with a decreased risk of gastroenteritis but not associated with allergic manifestations or lower respiratory tract infections [47]. A recent multicenter, randomized controlled trial comparing the outcomes for 657 preterm infants who consumed expressed breast milk from mothers taking either tuna oil (high DHA diet) or soy oil (standard DHA) capsules showed that DHA supplementation for infants of less than 33 weeks gestation reduced the incidence of bronchopulmonary dysplasia in boys and in all infants with birth weights less than 1250 grams [48]. DHA supplementation also reduced the incidence of reported hay fever in boys at either 12 or 18 months, which suggested a preventative role for respiratory allergy. However, the study did not result in reduction in the reported incidence of asthma, eczema, or food allergy [48]. 

## 4. Mechanisms by Which PUFAs Modulate Immune Function 

As pointed out earlier, the change in Western diets that consisted of relatively balanced ratios of omega-3 PUFAs and omega-6 PUFAs to a diet that was predominantly rich in omega-6 PUFAs has been suggested as a possible cause of high incidence of allergic diseases in the industrialized world [49]. Predisposition to allergic disease is postulated to result from insufficiently balanced T helper cell type 1 and 2 (Th1 and Th2) pathways during fetal life [50]. High concentration of dietary omega-6 PUFAs has been proposed to promote Th2 differentiation of the immune system during ontogeny and development [49]. Omega-3 PUFAs may alter the T helper cell balance by inhibiting interleukin-13 (IL-13) production, where IL-13 could be related to allergic diseases through its role in inducing IgE synthesis in B cells and Th2 type differentiation in T cells [51]. Thus, it is possible that diets high in omega-3 PUFAs may modulate the development of IgE mediated allergic diseases and regulate immune responses [34].

Diets rich in omega-6 fatty acids, through increased consumption of vegetable oils rich in LA, result in predominance of AA in tissues, which in turn gives rise to eicosanoids such as prostaglandin E2 [34]. Consequently, eicosanoids enhance the synthesis of Th2 cytokines and IgE antibodies, which is the hallmark of atopic responses to allergens [34]. Although it is beyond the scope of this paper to cover the mechanisms of PUFAs in modulating the immune system from the available literature, in this section, we highlight how PUFAs may exert its actions by modifying the cellular membrane, inducing eicosanoid metabolism and alerting gene expression.

### 4.1. Cellular Membrane Alteration

Omega-3 PUFAs from the diets can be incorporated into the membranes of essentially all cells, displacing AA, which leads to membrane modulation, affect lipid-protein interactions, and membrane lateral organization [52]. Biochemical and immunological changes, including alteration of receptor expression, reduction of prostaglandin E2 synthesis, and reduced proinflammatory cytokine responses can occur [10, 53]. Incorporation of PUFAs into antigen-presenting cells has been reported to downregulate their function and alter recognition by T cells [54]. EPA and DHA incorporate into lymphocyte membranes and alter the fluidity, suppress signal transduction and affect T-cell proliferation [55]. Furthermore, it has been shown to change the protein composition of the inner membrane lipid leaflet resulting in inhibition of T-cell responses and activation-induced cell death [55, 56]. 

### 4.2. Eicosanoid Metabolism through Competition between Omega-6 and Omega-3 PUFAs

Dietary omega-3 PUFAs also modify the fatty acid composition of membrane phospholipids by decreasing AA and increasing EPA which suppress eicosanoids associated with systemic inflammatory response syndrome. Eicosanoids are twenty carbon lipid mediators of inflammation that include prostaglandins (PGs), thromboxanes (TXs), leukotrienes (LTs), and other oxidized derivatives [12]. Phospholipase A2 cleaves membrane phospholipids to release AA which serves as a substrate for cyclooxygenase (COX) and lipoxygenase (LOX) enzymes leading to the production of eicosanoids [53]. Both COX and LOX enzymes are expressed in epithelial and inflammatory cells which give rise to different types of mediators [57, 58]. Presence of eicosanoid mediators can regulate the severity and length of inflammatory responses where some eicosanoids such as PGE2 are reported to play a role in promoting sensitization to allergens through actions on dendritic cells, T-cell differentiation, and Ig class switching in B cells [12]. In addition to proinflammatory effects, eicosanoids such as PGE2 have been reported to influence the Th1/Th2 balance, where PGE2 decreases the production of the Th1-type cytokines interferon (IFN-gamma) and IL-2, enhances the production of Th2-type cytokines IL-4 and IL-5, and promotes IgE synthesis by B cells [59, 60]. These eicosanoids are strongly associated with clinical manifestations of allergic diseases through their actions on inflammatory cells, smooth muscles, and epithelial cells [12].

Omega-3 PUFAs can exert immunosuppressive effects by competing with AA as substrates for COX and LOX enzymes, which in turn inhibit AA metabolism to lower the production of proinflammatory eicosanoids. Omega-3 PUFAs can also generate novel eicosanoids that have anti-inflammatory properties [61]. Interestingly, other omega-6 PUFAs were also found to exert anti-inflammatory effects [62], where the omega-6 PUFA dihomo-*γ*-linolenic acid (DGLA) can act as a competitive inhibitor of eicosanoid metabolism and inhibit the production of proinflammatory cytokines [63]. 

### 4.3. Gene Expression

It has been reported that PUFAs alter gene expression by either affecting signaling pathways or directly by interacting with nuclear receptors [64]. Transcription can be modified as PUFAs interact with sterol regulatory element binding proteins, liver X receptor, and peroxisome proliferator activated receptors (PPARs). PPARs are ligand-activated transcription factors present in a variety of cell types including inflammatory cells [65]. The omega-3 PUFAs are natural ligands of nuclear receptors such as peroxisome proliferator activated receptors PPAR-alpha and PPAR-gamma. The omega-3 PUFAs bind to PPAR-gamma, which has been shown to be involved in regulation of immune and inflammatory responses [66]. 

The omega-3 PUFAs also directly alter gene expression by modifying transcription factor activity such as nuclear factor-kB (NF-kB) via inhibition of the inhibitory subunit of NF-kB [10]. In response to inflammatory stimuli, NF-kB can modulate a range of inflammatory genes including COX-2, ICAM-1, VCAM-1, E-selectin, tumor necrosis factor-alpha, IL-1-beta, inducible nitric oxide synthase (iNOS), and acute phase protein [10]. The omega-3 PUFAs can influence the expression of cell adhesion molecules such as intercellular adhesion molecule-1 (ICAM-1), vascular cell adhesion molecule-1 (VCAM-1), E-selectin which in turn will direct the leukocyte-endothelium interactions, transendothelial migration of leukocytes, and trafficking of leukocytes [67].

## 5. Summary and Perspectives

Intake of proinflammatory omega-6 PUFAs has increased over the second half of the twentieth century, coinciding with increased prevalence of allergy and its clinical manifestations. Dietary sources of omega-3 PUFAs such as fish and fish oils can act to suppress the actions of omega-6 PUFAs, where the omega-3 PUFAs may protect against atopic sensitization. Studies investigating the effect of maternal fish intake during pregnancy on atopic or allergic outcomes in infant/children have demonstrated protective effects of omega-3 PUFAs against allergic diseases. However, further studies of increased omega-3 PUFAs consumption during pregnancy, lactation, and infancy are needed to better elucidate the immunologic and clinical effects and to identify protective or therapeutic effects. To date, evidence presented in this paper suggests that dietary intake/supplementation of omega-3 PUFAs during pregnancy may have greater impact on decreasing prevalence and severity of allergies in infants in comparison to dietary omega-3 PUFAs intake during lactation or directly to infants. There is also more convincing data on the benefits of providing omega-3 PUFAs in the form of fish compared to fish oils. Further studies are needed to determine the critical period of supplementation, as well as to compare if fish provide added benefits due to the accompanying nutrients when consumed together with fish oil. 

A few studies have investigated the impact of omega-3 PUFAs supplement on the risk of infections; however, the available literature seems to be limited to infections related to respiratory diseases [44, 46, 48]. Data from these studies are encouraging, indicating that there are benefits of omega-3 PUFAs supplementation in reducing the incidence of infectious respiratory diseases. These results may be particularly useful as a basis for potential clinical application of omega-3 PUFAs in reducing the risk of infection on preterm and intensive care unit infants. Data from various studies [34, 51] also indicate that omega-3 PUFAs may influence the activity of certain types of cells, which may subsequently affect the maturation and polarization of the immune system. Supplementation of the maternal diet and/or early childhood with omega-3 PUFAs may provide noninvasive intervention in possibly preventing other immune-mediated disease. Nevertheless, further investigations are warranted to assess the long-term effects of omega-3 PUFAs on the later immune-defense and health status during early growth and development.

## Figures and Tables

**Table 1:** Summary of studies on the effects of dietary PUFAs supplementation on allergic and respiratory diseases.

Study	Design		Subjects		Type of supplementation		Effects on	
		Types	age (months)	*n* =		Allergy	respiratory infections	Others
Morales et al., 2011 [47]	Cohort	Infants	0–14	580	Predominantly breastfed for 4–6 months	Protection against allergic manifestations—wheezing (adjOR = 0.53, 95% CI 0.32–0.89) and atopic eczema (adjOR = 0.58; 95% CI 0.32–1.04) between 7 and 14 months.	Significantly lower risk of lower respiratory tract infection (LRTIs) between 7 and 14 months (adjOR = 0.51, 95% CI 0.31–0.83) and for recurrent LRTIs (adjusted OR = 0.48, 95% CI 0.24–0.96).	(1) Reduced risk of gastroenteritis (GE) during first 6 months and recurrent GE (2) Exposure to higher doses of AA, DHA, and total *n*-3 associated with reduced risk of GE.

Manley et al., 2011 [48]	Randomized controlled trial	Preterm infants less than 33 weeks gestation	0–18	657	Breast milk from mothers taking either tuna oil (high-DHA diet) or soy oil (standard-DHA) capsules	(1) Reduction in reported hay fever in all infants in the high-DHA group at either 12 or 18 months (relative risk RR = 0.41, 95% CI 0.18–0.91; *P* = 0.03) in boys (RR = 0.15, 95% CI 0.03–0.64; *P* = 0.01) (2) No effect on asthma, eczema, or food allergy	Reduction in bronchopulmonary dysplasia in boys (RR = 0.67, 95% CI 0.47–0.96; *P* = 0.03) and in all infants with a birth weight of less than 1250 grams (RR = 0.75, 95% CI 0.57–0.98; *P* = 0.04)	No effect on duration of respiratory support, admission length, or home oxygen requirement

Sampath and Ntambi 2005 [44]	Randomized controlled trial	Children	0–36	89	DHA/AA supplemented formula (*n* = 38) versus nonsupplemented (*n* = 51) during the first year of life	DHA/AA group had significantly lower odds of having wheezing/asthma (OR = 0.31, 95% CI 0.10–0.90; *P* = 0.03), wheezing/asthma/AD (OR = 0.29; 95% CI 0.12–0.72; *P* = 0.008), or any allergy (OR = 0.30; 95% CI 0.12–0.73; *P* = 0.008) during the first 3 years of life compared with the control group	(1) DHA/AA group had significantly lesser episodes of upper respiratory infections (OR = 0.32; 95% CI 0.14–0.75; *P* = 0.008) (2) In addition, there was a tendency towards a lower number of episodes of combined nonallergic respiratory illnesses in the DHA/AA group (*P* = 0.06)	—

Grimm et al. 2002 [46]	Randomized controlled trial	Children	18–36	86	1st group—DHA 0 mg (*n* = 28) 2nd group—DHA 43 mg (*n* = 29) 3rd group—DHA 130 mg (*n* = 29)	—	Difference in respiratory illnesses detected between the groups (DHA-0 mg: *n* = 13, 46%; DHA-43 mg: *n* = 12, 41%; DHA-130 mg: *n* = 5, 17%; *P* = 0.039) with number of participants with events significantly lower in the DHA-130 mg versus DHA-0 mg group (*P* = 0.024)	Subjects consuming DHA-130 mg had significantly fewer adverse events than those consuming DHA-0 mg (*P* = 0.007)

Valledor and Ricote 2004 [45]	Randomized controlled trial	Healthy, nonbreastfed infants more than 36 weeks gestation	0–12	1342	DHA supplemented formula (*n* = 1094) and control group (*n* = 248)	—	(1) Significantly higher incidence of bronchiolitis/bronchitis observed in the control group compared to the DHA group at 5 months (13.9% versus 6.1%, *P* = 0.0001), 7 months (10.8% versus 5.1%, *P* = 0.01), and 9 months (11.3% versus 5.8%, *P* = 0.01) (2) Significantly higher occurrence of rhinitis at 1 month for the control group compared with the DHA group (6.7% versus 3.0%, *P* = 0.005) (3) Higher incidence of upper airway infection in the control group versus the DHA group at 1 month (12.1% versus 6.6%, *P* = 0.05) and 12 months (24.2% versus 16.2%, *P* = 0.01)	—
